# Magneto‐Based Synergetic Therapy for Implant‐Associated Infections via Biofilm Disruption and Innate Immunity Regulation

**DOI:** 10.1002/advs.202004010

**Published:** 2021-01-31

**Authors:** Jiaxing Wang, Lingtian Wang, Jiong Pan, Jinhui Zhao, Jin Tang, Dajun Jiang, Ping Hu, Weitao Jia, Jianlin Shi

**Affiliations:** ^1^ Department of Orthopaedics Shanghai Jiao Tong University Affiliated Sixth People's Hospital Shanghai Jiao Tong University Shanghai 200233 China; ^2^ School of Chemical Science and Engineering Tongji University Shanghai 200092 China; ^3^ Department of Clinical Laboratory Shanghai Jiao Tong University Affiliated Sixth People's Hospital Shanghai Jiao Tong University Shanghai 200233 China; ^4^ State Key Laboratory of High Performance Ceramics and Superfine Microstructure Shanghai Institute of Ceramics Chinese Academy of Sciences Shanghai 200050 China

**Keywords:** immunotherapy, implant‐associated infections, magnetic nanoparticles, magneto‐based synergetic therapy, nitric oxide

## Abstract

Implant‐associated infections (IAIs) are a common cause of orthopedic surgery failure due to microbial biofilm‐induced antibiotic‐resistance and innate immune inactivation. Thus, the destruction of microbial biofilm plays a key role in reducing IAIs. Herein, first, a magneto‐based synergetic therapy (MST) is proposed and demonstrated against IAIs based on biofilm destruction. Under an alternating magnetic field (AMF), CoFe_2_O_4_@MnFe_2_O_4_ nanoparticles (MNPs), with a rather strong magnetic hyperthermal capacity, can generate sufficient thermal effect to cause dense biofilm dispersal. Loosened biofilms provide channels through which nitrosothiol‐coated MNPs (MNP‐SNOs) can penetrate. Subsequently, thermosensitive nitrosothiols rapidly release nitric oxide (NO) inside biofilms, thus efficiently killing sessile bacteria under the magnetothermal effect of MNPs. More importantly, MNP‐SNOs can trigger macrophage‐related immunity to prevent the relapse of IAIs by exposing the infected foci to a consistent innate immunomodulatory effect. The notable anti‐infection effect of this nanoplatform is also confirmed in a rat IAI model. This work presents the promising potential of combining magnetothermal therapy with immunotherapy, for the effective and durable control and elimination of IAIs.

## Graphical abstract

This work originates a magneto‐based synergetic therapy (MST) strategy against implant‐associated infections (IAIs) based on nitrosated mercaptosuccinic acid (MSA)‐coated CoFe_2_O_4_@MnFe_2_O_4_ nanoparticles (MNP‐SNO) with excellent magnetic hyperthermal capacity, nitric oxide (NO) releasing efficiency, and innate immunomodulatory activity, which demonstrates the great potentials of magnetic nanomedicine for the durable control and elimination of IAIs.

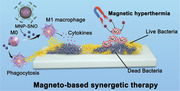

As a disastrous complication in orthopedics, implant‐associated infections (IAIs) are characterized by microbial biofilm formation both on indwelling devices and surrounding tissues, generally demanding multiple subsequent surgeries and huge costs.^[^ ^1^ ^]^ Once bacterial biofilms are formed, the dense biofilm matrix containing exopolysaccharides, proteins, and extracellular DNA shows increased capability of antibiotic resistance.^[^ ^2^ ^]^ In addition, biofilms inhibit host local innate immunity by switching proinflammatory macrophages (M1) to an anti‐inflammatory phase (M2), thus suppressing the phagocytic and bactericidal activities of macrophages.^[^ ^3^, ^4^, ^5^, ^6^, ^7^, ^8^ ^]^ To avoid recalcitrant biofilm‐associated infections, various antibacterial nanomaterials have been developed; these can be divided into either active therapeutic agents, with inherent anti‐infection features, or specialized delivery systems designed to release antiseptic drugs.^[^ ^9^, ^10^ ^]^ Although these active antimicrobial agents are often designed therapeutically to prevent biofilm formation on implants, concerns exist regarding both their potential cytotoxicity and long‐term stability.^[^ ^10^, ^11^ ^]^In addition, dead bacteria presented on the coating may further weaken the antibacterial activity of the contact‐killing surfaces of implants.^[^ ^12^ ^]^ Drug delivery nanosystems exhibit an initial burst‐release, followed by slow‐release of loaded drugs. Such a long‐time process of drug release will undoubtedly result in the drug resistance of bacteria.^[^ ^10^, ^13^, ^14^ ^]^ Consequently, it is still difficult for most reported nanotechnologies to destroy complex biofilm structure and eradicate mature IAIs. Moreover, few of these available nanomaterials are involved in improving the local immune microenvironment of IAIs. Thus, development of a novel nanoplatform that can thoroughly conquer implant‐associated infections through mature biofilm disruption and proinflammatory immunity stimulation would be of great significance.

Recently, magnetic nanoparticles with good biocompatibility are considered as a new approach to combat infection.^[^ ^15^, ^16^ ^]^ The magnetic field‐induced magnetic nanoparticles movement results in biofilm penetration and subsequently enhances the killing effects of antimicrobials.^[^ ^17^, ^18^ ^]^ Hyperthermia has also received increased attention lately, with its prominent antiseptic activity in damaging rigid biofilms.^[^ ^19^, ^20^ ^]^ Unlike photothermal therapy (PTT) that suffers from the limited depth of light penetration,^[^ ^21^ ^]^ magnetic hyperthermia (MH) is induced via alternating magnetic fields (AMFs) to avoid the limited tissue penetration,^[^ ^22^ ^]^ suggesting its optimal candidacy for treating deep IAIs in orthopedics. However, to thoroughly devitalize bacteria, a temperature above 80 °C must be sustained for 15 min, which will unfortunately destroy the surrounding normal tissues that can only withstand a few minutes of moderate heating at around 50 °C.^[^ ^23^, ^24^ ^]^ Therefore, it is hard to cure deep IAIs using single MH therapy, and the combing treatment involving other remedies is highly required.

Nitric oxide (NO), a kind of endogenous gas molecule with a broad antimicrobial spectrum, has been shown to penetrate deep into the biofilm to damage bacteria via the generation of reactive nitrogen species.^[^ ^20^ ^]^ Hence, NO molecule has attracted intense attention due to its potential as a bactericidal agent.^[^ ^24^, ^25^, ^26^, ^27^ ^]^ S‐nitrosothiols (RSNO), a thermosensitive NO donator,^[^ ^28^ ^]^ could be triggered to release NO to assault bacteria under hyperthermia. So it is desirable to destruct dense biofilm matrix and attack bacteria by MH and simultaneously released NO from prepared RSNO‐coated magnetic nanoparticles. Although the synergetic therapy of MH and NO is able to obliterate most bacteria inside biofilms, however, surviving pathogens in the infection focus may still evoke IAIs recurrence, especially in the biofilm‐caused disabled innate immunity area.^[^ ^29^, ^30^ ^]^ To address this serious issue, implants with immunoregulatory coatings (e.g., loaded with cobalt and copper) have been designed to switch macrophages from the M2 phase to M1 phase, which can successfully root out IAIs.^[^ ^31^, ^32^ ^]^ However, host proteins or dead bacteria covering the immunomodulatory surface may inhibit further antimicrobial immunity in vivo.^[^ ^12^ ^]^ Interestingly, it has been reported that magnetic nanoparticles not only promote inflammation when cocultured with macrophages, but also recruit macrophages,^[^ ^33^, ^34^ ^]^ with the proinflammatory response induced via the activation of the Toll‐like receptor pathway.^[^ ^35^ ^]^ As a kind of excellent immunomodulator, native magnetic nanoparticles themselves could both recruit additional macrophages and act as immunomodulators, thus promoting macrophage‐related immunity to deracinate deep IAIs.

Based on the magnetic nanoparticles therapeutic platform, we first propose magneto‐based synergetic therapy (MST) against IAIs, through which physical heating is utilized to achieve magnetothermal therapeutics. First, core–shell structured CoFe_2_O_4_@MnFe_2_O_4_ (abbreviated as MNP), with a magnetically hard CoFe_2_O_4_ core and soft MnFe_2_O_4_ shell, shows prominent MH performance due to their exchange‐coupled magnetism, and has been synthesized and modified by coating a layer of thermosensitive nitrosated mercaptosuccinic acid (MNP‐SNO).^[^ ^36^ ^]^ Furthermore, with remedial agent of NO released under AMFs from the nanosystem, bacteria inside the loosened biofilms would be damaged, and immunomodulatory antibacterial effects could be achieved via MNPs themselves. Schematically as shown in **Scheme** **1**, these obtained MNP‐SNO could generate AMF‐induced hyperthermia and simultaneously release NO gas to damage biofilms in vivo. Synergistically, macrophage‐related innate immunity is also stimulated by MNPs themselves, resulting in a sustained anti‐infection effect for biofilm elimination.

**Scheme 1:**
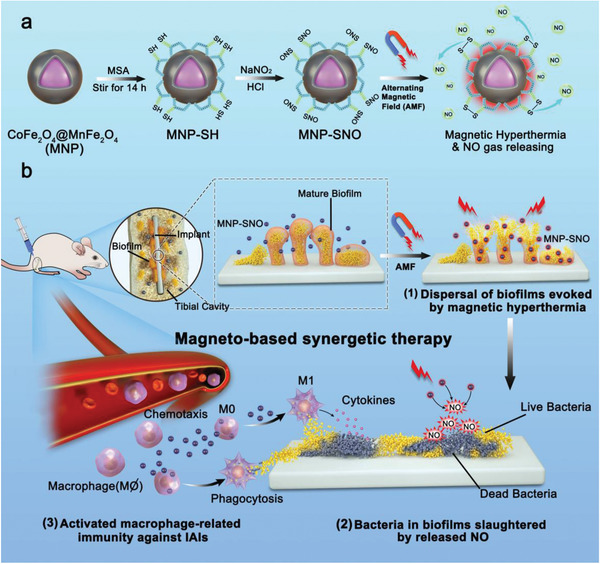
Schematic illustration of the synthesis of MNP‐SNOs and its in vivo magneto‐based synergetic therapy. a) Schematics illustrating the ligand exchange and nitrosation procedures of magnetic nanoparticles (CoFe_2_O_4_@MnFe_2_O_4_), and the concurrent magnetic hyperthermia/NO gas production under the applied alternating magnetic field (AMF). b) Schematics illustrating a rat tibia with infected implant under the treatments by MNP‐SNOs under AMF. 1) The mature and compact biofilms are fragmentized under magnetic hyperthermia by MNP‐SNOs, and 2) the abundant NO released from MNP‐SNOs in a hyperthermal environment is lethal to sessile bacteria inside biofilms. The MNP‐SNOs also act as chemokines and immunoregulators to direct macrophages to the infection focus and evoke M1 polarization of macrophages. 3) The surviving bacteria are mostly killed by phagocytosis and cytokine secretion of M1 macrophages.

The MNPs were synthesized using the thermal decomposition method (see the Experimental Section in the Supporting Information for the details of synthesis), with low magnification transmission electron microscope (TEM) images showing uniform size distribution and excellent dispersion of the nanoparticles (**Figure** **1**a). According to dynamic light scattering (DLS), the mean size of MNPs is 12.49 ± 1.97 nm. High‐resolution TEM images (Figure 1b; Figure S1, Supporting Information) exhibit MNP polycrystallinity, while the core–shell structure is confirmed using energy‐dispersive spectroscopy (EDS), as shown in Figure 1c. The merged image in Figure 1c clearly shows CoFe_2_O_4_ core/MnFe_2_O_4_ shell structures, with line‐scanning EDS profiles further verifying this structure (Figure S2, Supporting Information). Mercaptosuccinic acid (MSA)‐coated nanoparticles were originally designed as NO carriers to treat tumors,^[^ ^37^, ^38^ ^]^ with their bactericidal potential never being explored. Therefore, MNPs obtained in the oil phase were further modified with thiols from MSA using ligand exchange. The MSA‐coated MNPs (MNP‐SHs) intermediates were transferred into the aqueous phase for further modification. To confirm successful ligand exchange, infrared (IR) spectroscopic measurements were performed on MNPs and MNP‐SHs (Figure S3, Supporting Information). The IR‐spectrum of MNP‐SHs shows C—S and —COO— stretching vibrations at 1100 cm^−1^ (broad) and 1600 cm^−1^, respectively, which belong to thiol and carboxyl groups on MSA. Spectrum peaks at 3000 and 1500 cm^−1^ were ascribed to C=C and —CH_2_— bonds in oleic acid. Finally, MNP‐SHs were nitrosated with nitrous acid into MNP‐SNOs, which sustainably releases a low dose of NO at room temperature (Figure 1h). The mean size of MNP‐SNO was measured using DLS to be 70.88 ± 25.05 nm (Figure 1d), which is favorable for medical use.

**Figure 1:**
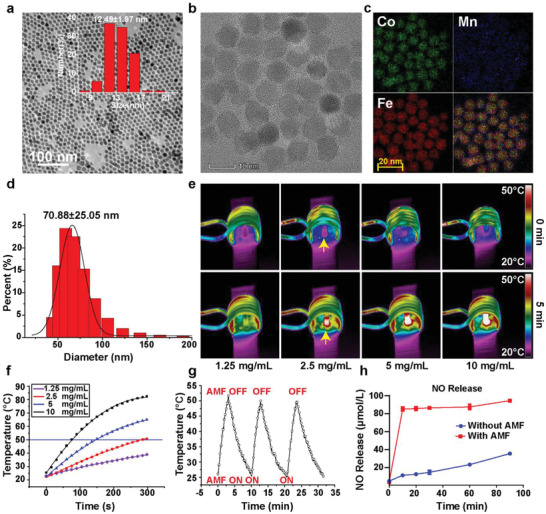
Characterization and property of MNP‐SNOs. a) Low‐resolution TEM image of CoFe_2_O_4_@MnFe_2_O_4_ nanoparticles (scale bar: 50 nm) and b) the enlarged image (scale bar: 10 nm). c) EDS mapping images of the MNPs. d) Particle size distribution curve of MNP‐SNOs suspended in water by DLS technique. e) Thermal images of MNP‐SNO solutions at varied concentrations under 1.35 kA m^−1^ AMF and f) the corresponding temperature–time curves. The yellow arrows in (e) point the background‐color area of MNP‐SNO (2.5 mg mL^−1^) before and after AMF application. g) Cyclic temperature–time curve of MNP‐SNO solution under AMF. h) NO gas release profiles of MNP‐SNOs with or without the magnetic hyperthermia stimulation.

Following phase transition and nitrosation, magnetic thermal properties were tested under an alternating magnetic field. Solutions of various MNP‐SNO concentrations were applied into a water‐cooled coil under a 1.35 kA m^−1^ AMF, and the temperature–time curves were recorded using an infrared camera. Thermal images in Figure 1e show that temperature of MNP‐SNO solution changes before and after AMF, while a decreased heating rate at reduced nanoparticle concentrations is found (Figure 1f). Generally, MNP‐SNO solutions at concentrations higher than 2.5 mg mL^−1^ could be heated above 50 °C in 5 min. Additionally, cyclic heating performance could be achieved by switching AMF on/off (Figure 1g), indicating the reversible magnetothermal effect of MNP‐SNOs in AMF. Importantly, magnetic hyperthermia induced a bursting release of more than 80 µmol L^−1^ NO in 10 min of heating (Figure 1h). In summary, MNP‐SNOs could generate both considerable amounts of heat and quick NO release in AMF.

Three kinds of cells, human foreskin fibroblast‐1 (HFF‐1), murine macrophage (RAW264.7), and murine preosteoblasts (MC3T3‐E1), were employed to evaluate the cytotoxicity of MNP‐SNO. Following 24 h coculture with different nanoparticulate concentrations, 5 mg mL^−1^ of MNP‐SNO shows some toxicity to HFF‐1 cells (Figure S4a, Supporting Information). Although differences in cellular morphology are nonsignificant among these groups, fluorescent staining of HFF‐1 (Figure S4b, Supporting Information) suggests that the number of adherent cells is decreased with the increase of MNP‐SNO concentrations, especially at 5 mg mL^−1^. Thus, 2.5 mg mL^−1^ was used as the optimal therapeutic dose to maintain both competent magnetic thermal performance and consistent NO loading. To explore cellular damage by MNP‐SNO under MH, 2.5 mg mL^−1^ MNP‐SNOs solution was cocultured with RAW264.7 cells and murine preosteoblasts (MC3T3‐E1), respectively. Cell culture disks were then performed with 1.35 kA m^−1^ AMF for 10 min. As shown in Figure S4c,d in the Supporting Information, compared to control group (treated with PBS), cell viability of the MNP‐SNO + MH group is still rather low in 24 h of proliferation, suggesting that MH originally causes large scale cell death. Then, MH‐induced cell death was partly erased in 48 h of cultivation. Moreover, survived cells from control and MNP‐SNO + MH groups after 48 h were further collected and seeded in new plates with the same cell amounts to evaluate the proliferation ability of these survived cells. Figure S4e,f in the Supporting Information illustrate that after culture for additional 24, 48, and 72 h, the proliferation rates of RAW264.7 and MC3T3‐E1 cells have no significant difference between the control and MNP‐SNO + MH groups, indicating that cells treated by NO and magnetic hyperthermia still have normal proliferative activity.

To confirm magnetic hyperthermia‐induced disruption of 3D biofilm barrier, biofilms cultured on confocal disks were divided into control (treated with saline), MNP‐SNO, MNP‐SH + MH, and MNP‐SNO + MH groups, with the two MH groups being treated under 1.35 kA m^−1^ AMF for 10 min. Treating temperature was held at 50 °C for ≈5 min, which is a widely utilized thermal condition during therapy.^[^ ^19^, ^23^, ^39^, ^40^, ^41^, ^42^ ^]^ Following the treatment, biofilms were stained with crystal violet (**Figure** **2**a). The quantitative results show that compared with the control and MNP‐SNO groups, MNP‐SH + MH and MNP‐SNO + MH groups evoke efficient biofilm dissociation and integrity destruction (Figure 2b; *n* = 3, *P* < 0.01). No significant differences are found between MNP‐SH + MH and MNP‐SNO + MH groups, suggesting that magnetic heating, instead of NO gas, dominates established biofilm disruption. Bacteria detached from the biofilm were also collected and examined under 490 nm absorbance^[^ ^43^ ^]^ (Figure 2c), and the results further illustrate that magnetic hyperthermia alone is powerful enough to dissociate mature biofilms, and release the bacteria therein. Biofilms were also treated with static magnetic field and water‐bath heating to reveal the main factor in resisting biofilms. Figure S5a,b in the Supporting Information indicates that high temperature plays a key role in biofilm disruption while magnetic force shows little effect. Moreover, biofilms cultured on shims were fixed and dehydrated for scanning electron microscopy (SEM) observation, which show a disintegrated biofilm in the MNP‐SH + MH group (Figure 2d), in comparison to the control group. The distribution of MNP‐SNO in the biofilm is characterized by a high‐resolution SEM (Figure S6, Supporting Information), and the result shows that the nanoparticles are distributed both on the surface (yellow arrows) and inside of the biofilms (red arrows), indicating some MNP‐SNO have been penetrated into biofilm after the dispersion (Figure S6a, Supporting Information). This result is also reflected in confocal laser scanning microscopy (CLSM) images, as well as a reconstructed 3D biofilm image (Figure 2e). The mean biofilm thickness in Figure 2e was quantified by ImageJ software (Figure 2f), and the thickness of the control group is 27.02 µm, which is significantly thicker than that of the MNP‐SH + MH group (16.16 µm, *n* = 3, *P* < 0.05), further confirming the destructive effect of magnetic hyperthermia on biofilm integrity. Figure S6b in the Supporting Information shows that numerous pores, as marked by red circles, are clearly visible in the confocal image of the biofilm after MH treatment, which may provide diffusing channels for MNP‐SNO nanoparticles to penetrate into biofilms.

**Figure 2:**
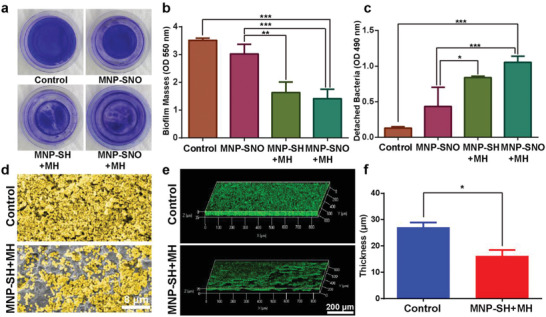
Biofilm disruption by magnetic hyperthermia. a) Macroscopic biofilm images of control (treated with saline), MNP‐SNO, MNP‐SH + MH (treated with 1.35 kA m^−1^ AMF for 10 min), and MNP‐SNO + MH (treated with 1.35 kA m^−1^ AMF and NO release for 10 min) groups; the bacteria were stained with crystal violet. b) Absorbance of biofilm masses at wavelength of 550 nm (*n* = 3, ^**^ *P* < 0.01 and ^***^ *P* < 0.001 using one‐way analysis of variance (ANOVA)). c) Absorbance of detached bacteria from biofilms at wavelength of 490 nm (*n* = 3, ^*^ *P* < 0.05 and ^***^ *P* < 0.001 using one‐way ANOVA). d) SEM images of biofilms from control and MNP‐SH + MH groups (scale bar: 8 µm). e) 3D‐reconstructed biofilms from the fluorescence images in Z‐stack in control and MNP‐SH + MH groups (scale bar: 200 µm). f) Quantification of 3D‐reconstructed biofilm thicknesses of control and MNP‐SH + MH groups as analyzed by ImageJ (*n* = 3, ^*^ *P* < 0.05 using the Student's *t*‐test).

Although hyperthermia is able to destroy mature biofilm, such short periods of mild magnetic hyperthermia therapy are ineffective to completely destroy biofilm‐like aggregates released from the dissociated biofilm, thus leading to possible biofilm regeneration. As a complementary bactericidal modality, NO gas was used to combine with mild MH treatment. As illustrated in **Figure** **3**, *Escherichia coli* and *Staphylococcus aureus* were used to confirm the synergic biofilm‐disruptive and antibacterial effects of MNP‐SNO. After counting bacterial colonies using the spread plate method (SPM), we found that, compared to control group (5.83 log_10_CFU mm^−2^) and MNP‐SNO group (5.73 log_10_CFU mm^−2^), both MNP‐SH + MH and MNP‐SNO + MH groups exhibit significant antibacterial efficacy (Figure 3b,c). The antibiofilm efficiency of the MNP‐SNO + MH group is much higher (3.34 log_10_CFU mm^−2^) than that of the MNP‐SH + MH group. The antimicrobial effects of different groups against bacteria inside biofilms are also qualitatively displayed in Figure 3a. These results demonstrate that slow NO release at room temperature or MH alone has very limited therapeutic effects against bacteria, while heat‐triggered NO release combined with MH induce a considerably stronger germicidal effect. In addition, to explore the dependence of biofilm elimination on MNP concentration, we chose the concentrations of 1.25, 2.5, and 5.0 mg mL^−1^ to treat the biofilms by the MNP‐SNO + MH treatment. As shown in Figure S7a,b in the Supporting Information, due to the insufficient thermal efficiency, the antibiofilm effect of the 1.25 mg mL^−1^ group was negligible, while 2.5 and 5 mg mL^−1^ groups exhibit prominent antibiofilm effect. Although the bacteriostatic effect of 5.0 mg mL^−1^ group is slightly better than 2.5 mg mL^−1^ group, however, the former (5.0 mg mL^−1^) induced higher cytotoxicity than the latter. Considering the bactericidal efficiency and biocompatibility, 2.5 mg mL^−1^ is chosen as the optimal concentration.

**Figure 3:**
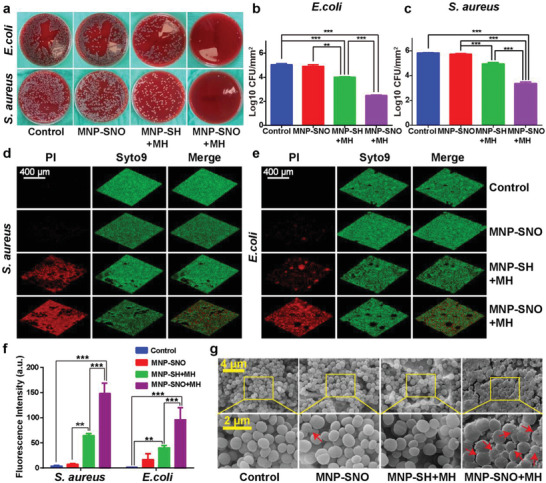
In vitro antibacterial assay of MH and NO therapies. a) Typical photos of *E. coli* and *S. aureus* biofilms treated with saline (control), MNP‐SNO, MNP‐SH + MH, and MNP‐SNO + MH groups (dilution rate: 1:10 000 for *E. coli*; 1:100 000 for *S. aureus*). Counting results of b) *E. coli* and c) *S. aureus* in four groups by a spread plate method (SPM) (*n* = 3, ^**^ *P* < 0.01 and ^***^ *P* < 0.001 using one‐way ANOVA). 3D‐reconstructions of the fluorescence labeled biofilms of d) *S. aureus* and e) *E. coli*, including PI signal (dead bacteria), Syto9 signal (all bacteria), and merged pictures (scale bar: 400 µm). f) Fluorescence intensity of red stained biofilms (dead bacteria) in four groups as analyzed by ImageJ (*n* = 3, ^**^ *P* < 0.01 and ^***^ *P* < 0.001 using one‐way ANOVA, Tukey's multiple comparisons test). g) High‐resolution SEM images of *S. aureus* biofilms attached on PEEK disks treated in different groups; the red arrows point out the distorted bacteria (scale bar: 4 and 2 µm).

To further reveal the antibiofilm or antisessile bacteria effects of different nanocomposites, dead and live bacteria of different groups were stained in red and green, respectively, as shown in Figure 3d,e. No red‐stained area can be seen in fluorescent images from control group (treated with saline), while few red fragments are found in the MNP‐SNO group. The biofilm structure is still intact, implying that a low dose of NO has a slight effect on biofilm integrality. In contrast, a thin layer of dead bacteria can be found in the MNP‐SH + MH group, suggesting that hyperthermia can destroy the biofilm and deactivate bacteria. However, the MNP‐SNO + MH group produces a completely intact red‐stained biofilm, while the green fluorescence reveals that the total amount of bacteria has also reduced significantly. Next, fluorescence intensity of red‐stained biofilms (dead bacteria) was analyzed with ImageJ, and the quantitative results demonstrate that bacterial death in the MNP‐SNO + MH group is considerably more significant than those in other groups (Figure 3f; *n* = 3, *P* < 0.01). Furthermore, we observed the morphology of treated biofilm using SEM, and found that most *S. aureus* maintained a spherical morphology with an integrated cell membrane in groups without NO release. In contrast, the combined application of magnetic hyperthermia and NO resulted in biofilm dispersal and distorted morphology of bacterial cells (Figure 3g). Similarly, *E. coli* treated with MNP‐SNO and MH also lose its original rod‐like morphology, showing a disrupted biofilm (Figure S8, Supporting Information).

Although magnetic hyperthermia could effectively damage biofilms, and released NO could kill majority of bacteria, IAIs could still recur. To solve this problem, MNP‐SNOs were designed to recruit macrophages and switch them to the M1 phase to further suppress IAIs. RAW264.7 macrophages were treated with either phosphate buffered saline (PBS), lipopolysaccharide (LPS), or MNP‐SNO, and then collected for flow cytometry analysis. The results show that, compared to the PBS group (negative control), CD86 fluorescence intensity is much higher in both LPS (positive control) and MNP‐SNO groups, revealing that M1 polarization of RAW264.7 has been dramatically promoted by MNP‐SNO (**Figure** **4**a,b). According to the literature, macrophage innate immunity against bacteria involves chemotaxis, secretion of inflammatory cytokines, and phagocytosis.^[^ ^44^ ^]^ Therefore, these indicators were chosen to identify macrophage polarization in subsequent immunoregulation experiments. As shown in Figure 4c, only MNP‐SNO‐treated cells can be stained with Perl's blue, suggesting that MNP‐SNO may stimulate proinflammatory polarization of macrophages (from M0 to M1) following the ingestion by RAW264.7 cells. Typical proinflammatory cytokines, tumor necrosis factor (TNF)‐*α* and interleukin (IL)‐1*β*, were also measured using an enzyme‐linked immunosorbent assay (ELISA) kit. In comparison to the control group, the levels of TNF‐*α* and IL‐1*β* can be found to be upregulated with MNP‐SNO stimulation (*n* = 3, *P* < 0.05) (Figure 4d). Furthermore, as a M2‐related anti‐inflammatory cytokine, levels of IL‐10 are relatively high in the control and LPS groups, while MNP‐SNO group displays a downregulated expression of IL‐10.

**Figure 4:**
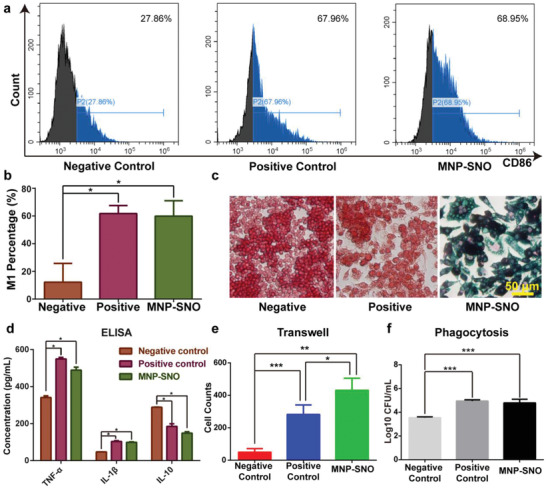
In vitro immunomodulation assay of magnetic nanoparticles. a) Flow cytometry results of RAW264.7 (CD86 is the marker for M1) from negative control (treated with 20 µL PBS for 24 h), positive control (treated with 10 µg L^−1^ LPS for 24 h), and MNP‐SNO (treated with 2.5 mg mL^−1^ MNP‐SNO for 24 h) groups, and b) the quantitative analysis of M1 macrophage percentage in three groups (*n* = 3, ^*^ *P* < 0.05 using one‐way ANOVA). c) Perl's blue staining images of RAW264.7 cells cocultured with PBS, LPS, or MNP‐SNO. The nuclei of macrophages were stained in red, and the iron element in cells was stained with Perl's blue (scale bar: 50 µm). d) ELISA results of cytokines (TNF‐*α*, IL‐1*β*, and IL‐10) secreted by RAW264.7 in different groups (*n* = 3, ^*^ *P* < 0.05 using one‐way ANOVA). e) Cell counts of the migrated RAW264.7 cultured on transwells (*n* = 3, ^*^ *P* < 0.05, ^**^ *P* < 0.01, and ^***^ *P* < 0.001 using one‐way ANOVA, Tukey's multiple comparisons test). f) Counted results of phagocytized *S. aureus* by RAW264.7 treated in different conditions (*n* = 3, ^***^ *P* < 0.001 using one‐way ANOVA).

Real‐time quantitative polymerase chain reactions (RT‐qPCRs) were also performed to examine the expression of TNF‐*α*, inducible nitric oxide synthase (iNOS, proinflammatory gene), and arginase (Arg‐1, anti‐inflammatory gene). As Figure S9 in the Supporting Information shows, TNF‐*α* and iNOS expressions in the positive control and MNP‐SNO groups are significantly upregulated, in comparison with the negative control group (*n* = 3, *P* < 0.01). While no distinctive difference of Arg‐1 expression can be found among all groups. Additionally, as shown in Figure 4e and Figure S10 in the Supporting Information, using the transwell assay, we can find that the MNP‐SNO group has greatly promoted RAW264.7 cell migration. This effect is consistent with the result from LPS positive group, while such a chemotaxis is not present in the negative control group. To verify the phagocytic function of activated M1 macrophages, RAW264.7 in three pretreated groups was cocultured with *S. aureus*. Phagocytized microbes were then collected from lysed macrophages and counted by spreading plate method (Figure S11, Supporting Information). LPS and MNP‐SNO groups clearly show enhanced phagocytic effects, while the number of phagocytized *S. aureus* in the negative control group is much lower (Figure 4f; *n* = 3, *P* < 0.05). These results demonstrate that MNP‐SNO is an excellent immunomodulatory reagent, which recruits macrophages and promotes M0 macrophage switching to the proinflammatory M1 phase. Previous reports indicate that bacterial biofilms could induce the anti‐inflammatory polarization (M2) of macrophages to depress local innate immunity.^[^ ^3^ ^]^ Encouragingly, MNP‐SNO is found to be capable of reactivating antibacterial functions of macrophage, thus reversing biofilm‐induced immune evasion.

The promising antibiofilm and immunomodulatory effects of MNP‐SNO under AMF encouraged us to cure deep implant‐associated infection in vivo (**Figure** **5**a). Before surgery, poly‐ether‐ether‐ketone (PEEK) implants were immersed in tryptic soy broth (TSB) medium containing *S. aureus* to form biofilms. Following implantation of a biofilm‐coated PEEK rod, rat tibias were injected with different groups of aqueous solutions. Next, the right legs of rats were exposed to 1.35 kA m^−1^ AMF for 10 min. The infrared camera was used to monitor and record the variation of temperature (Figure 5b). In the MNP‐SNO group, a rapid rise in temperature is observed at the focus, with the temperature maintained at 50 °C for ≈5 min (Figure 5c). In contrast, only a slight increase of temperature (from 32 to 40 °C) has been recorded in the control group (injected with saline). These results indicate that the magnetic field could penetrate and reach deep bone tissue, thus evoking the hyperthermia effect in vivo. In addition, an X‐ray radiogram was taken at varied time intervals to evaluate osteomyelitis progression. Both periosteal reaction and general impression in the radiographic images were assessed according to previous research.^[^ ^45^ ^]^ As shown in Figure S12 in the Supporting Information, bone destruction and depressed bone formation are visible in control, MNP‐SNO, and MNP‐SH + MH groups, while no significant infection was observed in MNP‐SNO + MH group.

**Figure 5:**
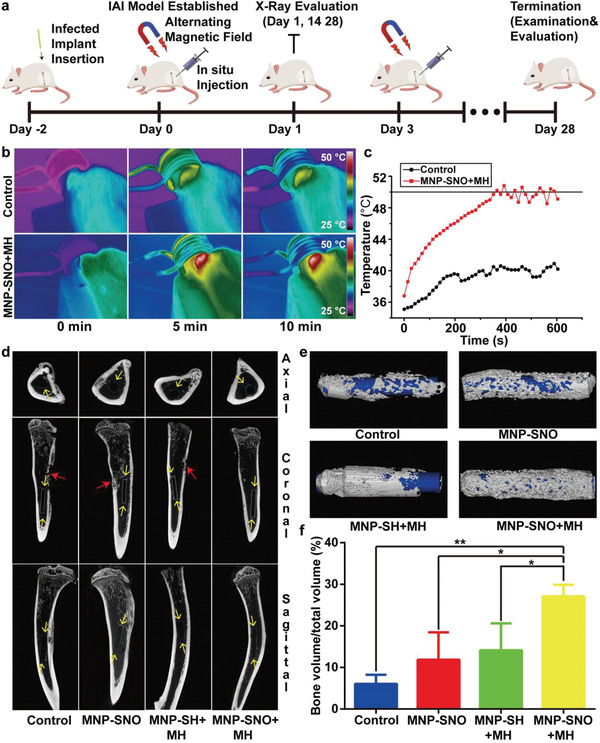
In vivo treatments and radiological evaluation. a) Scheme of deep IAI model establishment and subsequent treatment, examination, and evaluation at different time points. b) Thermal images of the control (injected with saline) and MNP‐SNO + MH groups under AMF for 10 min in vivo, and c) the corresponding temperature–time curve. d) Micro‐CT images of the tibias from four groups (control, MNP‐SNO, MNP‐SH + MH, and MNP‐SNO + MH). The red arrows indicate the infected dead bones, and the yellow arrows point the implant and new bones. e) 3D reconstructions of new bones and implants in different groups. The white masses indicate the new bone, and the blue rods represent implants. f) Quantitative analysis of bone mass in four groups (*n* = 3, ^*^ *P* < 0.05 and ^**^ *P* < 0.01 using one‐way ANOVA, Tukey's multiple comparisons test).

To further confirm implant location and new bone reconstruction around it, we used micro‐computed tomography (CT) to scan the harvested tibias. In Figure 5d, as marked by yellow arrows, newly generated bone can be clearly identified in the axial, coronal, and sagittal planes in all groups. In contrast, no dead bones can be found for MNP‐SNO + MH group while other groups clearly exhibit dead bones. Figure 5e illustrates the 3D reconstruction of new bone and implants. Implants have been completely covered by new bone in the MNP‐SNO + MH group, while bone repairs are incomplete in the other groups. Moreover, the volume of new bone was also analyzed, and results in Figure 5f show no significant difference among the control, MNP‐SNO, and MNP‐SH + MH groups. In contrast, the bone volume/total volume ratio of the MNP‐SNO + MH group (27.11%) is much higher than those of other groups (*n* = 3, *P* < 0.05). According to a previous report, when IAIs occur, bacteria will strongly compete with osteoblasts on the surface of the implant, leading to inhibited osteogenesis with severe infections.^[^ ^46^ ^]^ Thus, results of the micro‐CT confirm that MNP‐SNO + MH can successfully disrupt preformed biofilms, thus providing a reliable environment for osteoblast adherence and growth.

To visualize the in vivo anti‐infective effects of nanocomposites, implants and ambient bone tissues were also harvested for microbial load derivation (**Figure** **6**a). As shown in Figure 6b, the groups of control, MNP‐SNO, and MNP‐SH + MH display large amounts of bacterial colonies on the agar, while only few separate colonies are present in the MNP‐SNO + MH group. In addition, implants were immersed in physiological saline, and treated with ultrasonic concussion to detach sessile bacteria. The amount of bacteria was then counted using spread plate methods. The corresponding results shown in Figure S13 in the Supporting Information) indicate that biofilms are predominantly eradicated in the MNP‐SNO + MH group. Furthermore, the total amount of bacteria in the surrounding bone tissue of MNP‐SNO + MH group has significantly decreased, compared to other three groups (Figure 6c,d; *n* = 3, *P* < 0.001).

**Figure 6:**
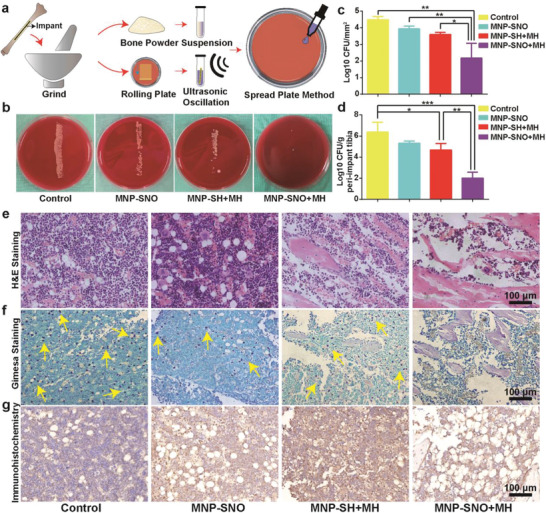
In vivo anti‐infection and immunoregulation evaluations. a) The processing scheme of tibias and implants from IAI rats. b) The bacterial colonies of roll‐over cultures from explanted PEEK rods when the in vivo experiment was terminated. c,d) Quantitatively analyzed SPM results of biofilms detached from implants and resident bacteria in surrounding tissues in different treated groups (control group: treated with saline). H&E and Giemsa staining images of tibias from different groups showing e) the degree of inflammation infiltration and f) the amounts of pathogens. Yellow arrows in (f) point the stained bacteria or their cluster (scale bar: 100 µm). g) Immunohistochemical images of tibias stained with iNOS antibody, with the stained areas being in brown (*n* = 3, ^*^ *P* < 0.05, ^**^ *P* < 0.01, and ^***^ *P* < 0.001 using one‐way ANOVA).

Interestingly, compared with the control group, either MH or NO single therapy has shown a definite bactericidal effect in vivo, which is more significant than that of their in vitro antibacterial effects. Hence, we suppose that the persistent immunomodulatory activity of MNPs plays an important role in preventing biofilm infection. Additionally, hematoxylin and eosin (H&E) and Giemsa staining were utilized to evaluate inflammation and bacterial residues at the lesions. A large number of inflammatory cells are observed in the control and MNP‐SNO groups, indicating that inflammation is still severe at 4 weeks post‐treatment. More new bone formation and less inflammatory infiltration are found in the MNP‐SH + MH and MNP‐SNO + MH groups, revealing that IAI could be controlled and bone tissue could be regenerated (Figure 6e). As illustrated by yellow arrows in Figure 6f, numerous bacteria are found in the control and MNP‐SNO groups, while only a few bacteria and no pathogen are observed in MNP‐SH + MH and MNP‐SNO + MH group, respectively. Immunohistochemical staining of iNOS, an M1 phase macrophage marker, was conducted to evaluate macrophage proinflammatory polarization. As presented in Figure 6g, no iNOS‐stained region is identified in the control group, revealing biofilm‐induced deterioration of local innate immunity (e.g., macrophages). However, considerably more M1 macrophages are discovered in MNP‐SNO, MNP‐SH + MH, and MNP‐SNO + MH groups, demonstrating that biofilm‐induced immune escape has been largely suppressed through the nanoparticlulate immunomodulatory effects, leading to largely sustained immunity for at least 4 weeks.

The degradation of MNP‐SNO in vivo was also explored. The nanoparticles in tibias sharply drop into 29.55% and 10.66% on day 7 and day 14, respectively, compared to that on day 1 (Figure S14, Supporting Information), while no MNP‐SNO is detected in other organs (heart, liver, spleen, lung, and kidney). This result suggests that MNP‐SNO can exist in tibias for more than 2 weeks to stimulate the M1 polarization and recruit macrophages. As the life span of macrophages is several months,^[^ ^47^ ^]^ thus the M1 polarization of macrophages can be easily maintained for another 2 weeks, which confers a persistent bactericidal effect. Organs, including hearts, livers, spleens, lungs, and kidneys, of rats from each group were harvested and analyzed. Results show no significant differences between groups, indicating that the nanoparticles were biosafe and nontoxic to animals (Figure S15, Supporting Information).

In summary, the induction of moderate magnetic hyperthermia via synthesized magnetic nanoparticles (MNP‐SNO) is able to disrupt the dense 3D biofilm structure. Furthermore, the magnetic heating‐induced bursting release of NO further destroys biofilm‐like aggregates. In addition, MNP‐SNOs themselves could efficiently recruit macrophages and actuate their polarization switching to the M1 state, thus activating local innate immunity against biofilms and resulting in a sustained and complete anti‐infective effect. Due to the high penetrability of alternating magnetic field, significant antibiofilm and persistent immunomodulatory effects of MNP‐SNOs have been verified in rat IAI models. As a promising prospect in the anti‐infection field of magnetic nanomedicine, our study provides a new strategy to treat deep IAIs by combined magnetothermal therapy and immunotherapy.

## Conflict of Interest

The authors declare no conflict of interest.

## Supporting information

Supporting Information
