# Supplementary material for: Magneto‐Based Synergetic Therapy for Implant‐Associated Infections via Biofilm Disruption and Innate Immunity Regulation
Source: Adv Sci (Weinh). 2021 Jan 31;8(6):2004010. doi: 10.1002/advs.202004010 (PMC7967068; doi:10.1002/advs.202004010)
Supplement: Supplementary file 1 — Supporting Information [file ADVS-8-2004010-s001.pdf]

© 2020 Wiley-VCH GmbH

ADVANCED SCIENCE
Open Access

Supporting Information

for *Adv. Sci.*, DOI: 10.1002/advs.202004010

Magneto-based Synergetic Therapy for Implant-Associated Infections via Biofilm Disruption and Innate Immunity Regulation

Jiaxing Wang, Lingtian Wang, Jiong Pan, Jinhui Zhao, Jin Tang, Dajun Jiang, Ping Hu, Weitao Jia*, and Jianlin Shi**

Supporting Information

Magneto-based Synergetic Therapy for Implant-Associated Infections via Biofilm Disruption and Innate Immunity Regulation

Jiaxing Wang[+], Lingtian Wang[+], Jiong Pan[+], Jinhui Zhao, Jin Tang, Dajun Jiang, Ping Hu[*], Weitao Jia[*], and Jianlin Shi[*]

Dr. J. Wang, L. Wang, J. Zhao, D. Jiang, Prof. W. Jia
Department of Orthopaedics, Shanghai Jiao Tong University Affiliated Sixth People's Hospital, Shanghai Jiao Tong University, Shanghai 200233, China

Prof. P. Hu, Prof. J. Shi
State Key Laboratory of High Performance Ceramics and Superfine Microstructure, Shanghai Institute of Ceramics, Chinese Academy of Sciences, Shanghai 200050, China

Dr. J. Pan
School of Chemical Science and Engineering, Tongji University, Shanghai 200092, China

Prof. J. Tang
Department of Clinical Laboratory, Shanghai Jiao Tong University Affiliated Sixth People's Hospital, Shanghai Jiao Tong University, Shanghai 200233, China

[+]These authors contributed equally to this work.

Experimental Section

***Materials*:** Ferric acetylacetonate, cobalt acetylacetonate, manganese acetylacetonate, and 1,2-hexadecanediol were purchased from Macklin Reagent Co. (China). MSA was obtained from Jiuding Chemical Reagent Co. (China). Sodium nitrite and ethylenediaminetetraacetic acid (EDTA) were purchased from Aladdin Reagent Co. (China). Griess Reagent was purchased from Sigma-Aldrich (Germany), and LIVE/DEAD™ BacLight™ Bacterial Viability Kit was purchased from Invitrogen (USA). Lipopolysaccharide (LPS) was obtained from Solarbio technology Co. (China). APC anti-mouse CD86 antibody was purchased from BioLegend (USA), while TNF-α，IL-1β, and IL-10 ELISA kits were purchased from Multi Science Biotech Co. (China). PEEK spacers (10 mm × 10 mm × 1 mm) and rods (6 mm length, 1 mm diameter) were purchased from Aosheng Materials Co. (China).

***Synthesis of $CoFe_2O_4$@$MnFe_2O_4$ nanoparticles*:** To synthesize MNPs, 0.5 mmol of ferric acetylacetonate, 0.25 mmol of cobalt acetylacetonate, 2.5 mmol of 1,2-Hexadecanediol, 1.5 mmol of oleic acid, 1.5 mmol of oleylamine, and 15 mL of dibenzyl ether were mixed and stirred for 15 min at 110 ºC, followed by 2 h of heating at 200 ºC. Next, the mixture was heated to boiling point (approximately 290 ºC) and refluxed for another 1 h. The procedure above was performed under a flow of helium. After 1 h of cooling, 50 mL of ethanol was added, and the mixture was then centrifuged. The resulting precipitate was dissolved in 10 mL of toluene. Finally, 50 μL of both oleic acid and oleylamine were added to the solution. Impurities were separated using centrifugation, and $CoFe_2O_4$ nanoparticles were obtained. To cover the core with a $MnFe_2O_4$ shell, 2 mL of $CoFe_2O_4$ solution was mixed with the initial reaction system where cobalt acetylacetone was substituted with manganese acetylacetone. The previously described steps were performed to construct $CoFe_2O_4$@$MnFe_2O_4$ nanoparticles (MNPs).

***Ligand exchange and thiol nitrosation of nanoparticles*:** First, oil phase MNPs (5 mg) were mixed with MSA (260 mg) dissolved in 1 mL of dimethyl sulfoxide (DMSO), and the mixture stirred fiercely for 24 h. Next, oleic acid coated on MNPs was replaced with MSA, and MNP-SH were formed and obtained using centrifugation. The precipitate was washed twice with ethanol and water, and water phase of MNPs were formed using ultrasonic oscillation. To complete thiol nitrosation on the MNP surface, 25 μL of hydrochloric acid (1.2 mol/L) and 200 μL of aqueous sodium nitrite (60 mmol/L) were added to 1 mL of MNP-SHs (5 mg), and incubated for 20 min at 4 ºC. After being repeatedly washed with water, MNP-SHs were transformed to MNP-SNOs.

Characterization: TEM images and element mappings were obtained using a ThermoFisher Talos F200X electron microscope (ThermoFisher, USA). MNP-SNO size distributions were measured using a Nicomp 380 submicron Particle Sizer (PSS, USA). Infrared spectra were performed using a Fourier transform infrared spectrometer (FTIR 850, Tianjin Gangdong SCI & Tech. Development Co., LTD). *In vitro* and *in vivo* magnetic thermal images were captured using Fotric 236 (FOTRIC Thermal Intelligence Co., China, MSX Resolution: 384 × 288; thermal sensitivity: <0.05 °C; temperature range: -20 °C to 650 °C, accuracy: ±2 °C).

Detection of NO: To detect the NO loading capacity and releasing profile of MNP-SNOs, 10 mg of nanoparticles were dissolved in aqueous solution and divided into two groups: one treated with AMF for 10 min, and the other kept at room temperature for 10 min. Next, MNP-SNOs were separated from solution using magnetic adsorption, and 50 μL of supernatant was collected and mixed with 50 μL of Griess reagent. Following 10 min incubation at 37 °C, NO content was measured using an enzyme-labeled instrument (BioTek Epoch, USA) at a wavelength of 540 nm. Finally, the NO concentration was measured after 10, 20, 30, 60, and 90 min.

In vitro cytotoxicity evaluation: HFF-1 and RAW264.7 cells were used to evaluate cytotoxicity. These two cell lines were incubated in high glucose Dulbecco’s modified Eagle’s medium (DMEM), with 10% fetal bovine serum, 100 U/mL penicillin, and 100 μg/mL streptomycin. Cells were seeded in 24-well plates at a concentration of 1×10^4 cells per well and cultured for 12 h at 37 °C. Stagnant medium was then replaced with fresh medium, containing different amounts of MNP-SNOs (0, 1.25, 2.5, 5 mg/mL). Following a 24 h incubation, medium with nanoparticles was replaced with 10% CCK-8 (Cell Counting Kit-8, Dojindo, Japan) reagent and incubated for 2 h. An enzyme-labeled instrument was then used to measure absorbance at 450 nm. To evaluate MNP-SNO+MH-caused damage, cell medium containing 2.5 mg/mL MNP-SNOs was cocultured with MC3T3-E1 and RAW264.7 cells (4×10^4 cells per disk) for 12 h, followed by 1.35 kAm^{-1} AMF for 10 min. After MH treatment, MC3T3-E1 and RAW264.7 cells were collected and seeded onto new disks. CCK-8 assays were performed every 24 h in MNP-SNO+MH and control groups. After culture for 48 h, cells in control and MNP-SNO+MH groups were further collected and seeded in new 24-well plates (1×10^4 cells per well) to determine the proliferation ability of the survived cells. Cell proliferation in the two groups were examined with standard cytometry after additional 24, 48, 72 h cultivation.

Furthermore, treated HFF-1 cells were fixed with a 4% formaldehyde solution and stained with fluorescein isothiocyanate (FITC) labeled phalloidin (FITC Phalloidin, Yeasen Biotechnology, China) and

4'6-diamidino-2-phenylindole (DAPI , Solarbio Biotechnology, China). Fluorescence stained cells were then observed under a CLSM (CLSM, ZEISS LSM 710, ZEISS, German).

***In vitro anti-biofilm structure and anti-infection assay*:** *S. aureus* (ATCC 43300) and *E. coli (ATCC 25922*) incubated in Tryptic Soy Broth (TSB) medium were applied to determine the potential of different nanocomposites against dense biofilm structure. Bacterial suspensions, with a concentration of 10^7 CFU/mL, were placed into confocal dishes in an incubator at 37 °C. The biofilms were formed on the surface of dishes after 72 h incubation. Next, TSB medium was replaced with physiological saline containing nanoparticles, thus exposing mature biofilms to either MNP-SNO (2.5 mg/mL), MNP-SH (2.5 mg/mL)+MH, or MNP-SNO (2.5 mg/mL)+MH, while the group without any treatment was regarded as control group. Next, supernatants in confocal dishes were transferred to a 96-well plate and analyzed using a wavelength of 490 nm. Furthermore, biofilms remaining at the bottom of confocal dishes were fixed with methanol and stained with crystal violet. The dye was then washed with 30% ethylic acid, and the eluent was measured using an enzyme-labeled instrument at a wavelength of 550 nm. Additionally, to disclose the main role of antibiofilm, mature biofilms were also treated with static magnetic field (overnight) and water heating bathe (from 37 °C to 50 °C, 10 min) respectively. For CLSM photography, established biofilms were also stained with propidium iodide (PI, 3 μL/mL) and Syto-9 (1: 1) in a dark room for 30 min. Biofilm thickness in each group was measured using ImageJ software. To further evaluate anti-biofilm effects, fluorescent intensity of PI channel from each groups was measured using ImageJ. For SEM (Zeiss evo18, German), biofilms were cultured on PEEK spacers. The mature biofilms were fixed with 2.5% glutaraldehyde for 24 h at 4 °C. Then, they were dehydrated using graded ethanol (30%, 50%, 70%, 80%, 90%, 95% and 100%) for 10 min at room temperature for each gradient. Following freeze drying and gold sputter coating, samples from four groups were observed by using SEM.

Meanwhile, *S. aureus* and *E. coli* were cultured into biofilms and treated with the same method described above. These treated biofilms were also divided into four groups: control, MNP-SNO, MNP-SH+MH and MNP-SNO+MH group. After different treatments, supernatants and biofilms were collected in 1 mL of physiological saline and ten-fold diluted into a series of gradients. Subsequently, 100 μL of these dilutions were extracted and spread onto sheep blood agar plates (SBA). Following overnight incubation at 37 °C, bacterial colonies on the plates were counted, and the antimicrobial effect of each group was compared. The experimental procedure described above is called the spread plate method (SPM).

Flow cytometry analysis and immunoregulation detection of macrophages**:** Murine RAW264.7 cells were seeded onto a 6-well plate at a concentration of 1×10^5 cells/well. After incubating for 12 h, 20 μL of PBS, 20 μg of LPS (10 μg/mL), and 5 mg of MNP-SNOs (2.5 mg/mL) were added into each well. Following 24 h incubation, RAW264.7 cells were scraped and collected using centrifugation at 500 rpm for 5 min. Cell masses were then resuspended with 100 μL of PBS containing 1 μg of Allophycocyanin (APC)-labeled CD86 antibody, and sequentially incubated on crushed ice for 30 min. Finally, CD86 expression on RAW264.7 macrophages was detected using the Beckman CytoFLEX system (Beckman Coulter, USA), with results being analyzed using CytExpert software.

To assess inflammatory cytokine expression, RAW264.7 cells were treated with the same method as described above, prior to collection and centrifugation of the cell medium at 3000 rpm for 20 min, to remove cell debris and nanoparticles. Expression levels of TNF-α, IL-1β, and IL-10 in the obtained supernatants were detected using ELISA kits, according to the manufacturer's protocol.

RAW264.7 cellular uptake of MNP-SNO nanoparticles was verified using Perl's blue staining (Yeasen Biotechnology, China). Pre-treated RAW264.7 cells in different groups were fixed with 4% (v/v) formaldehyde for 15 min, followed by Perl's blue staining for 30 min. Within 1 min of using nuclear fast red to stain the nucleus of macrophages, results were observed under an optical microscope (OLYMPUS, IX70, Japan).

RAW264.7 cell inflammatory-related gene expression was evaluated in negative control, positive control, and MNP-SNO groups, respectively. In brief, RAW264.7 cells were seeded onto a 6-well plate at a concentration of 1×10^5 cells/well, and treated using the method described above. After culturing for 24 h, TNF-α, iNOS and Arg-1 gene expression was analyzed using RT-qPCR. In brief, total RNA was extracted using an EZ-press RNA Purification Kit (EZBioscience), according to the manufacturer's instructions. Purified RNA was then reversely transcribed into cDNA using a Color Reverse Transcription Kit (EZBioscience). Next, RT-qPCR was performed using a 2 × Color SYBR Green qPCR Master Mix (EZBioscience) on a QuantStudio 7 Flex system (Life technologies). Gene expressions were calculated using the $2^{-\Delta\Delta Ct}$ method. Primer sequences for TNF-α, iNOS, and Arg-1 genes are shown in Table S1.

The chemokine-like function of MNP-SNO was confirmed using a transwell migration assay. Briefly, RAW264.7 cells (1×10^5 cells/sample) were seeded onto 8 μm transwells. Next, 2.5 mg/mL of MNP-SNOs were added into the lower chambers while LPS and PBS were also added into other two groups as a positive and negative control. Following incubation for 24 h, cells on the bottom of the transwell were stained with crystal violet and counted using optical microscopy.

Additionally, phagocytic activity of RAW264.7 cell was estimated in the negative control, positive control, and MNP-SNO groups, respectively. Briefly, RAW264.7 cells were seeded onto 6-well plate at a concentration of 2×10^5 cells/well, and treated using the aforementioned method. Next, the gentamicin protection assay was performed, according to the literature.[1, 2] Briefly, following incubation for 24 h at 37 °C, supernatant was removed and DMEM containing 10^7 CFU/mL of *S. aureus* was added into each well for 30 min coculturing with RAW264.7 cells. Next, the bacterial suspension was replaced with fresh DMEM containing 200 μg/mL of gentamicin, and cultivated for another 1 h to damage extracellular bacteria. After removing the antibiotic solution and washing macrophages with PBS, 1 mL of 1% Triton X-100 was added to each well to penetrate the cell membrane of macrophages, thus inducing engulfed bacteria leakage. Triton X solution was collected to enumerate the bacteria by gradient dilution and spreading plate method.

In vivo anti-infection and immunoregulatory assay

Implant-related tibial osteomyelitis model**:** Specific-pathogen-free Sprague Dawley rats (350-400 g) were purchased from the School of Agriculture and Biology, Shanghai Jiao Tong University. The animal experiment was approved by the Animal Ethical and Welfare of Shanghai Jiao Tong University Animal Department and the approval number is 20191230. Forty male rats were divided into the following four groups: Control, MNP-SNO, MNP-SH+MH, and MNP-SNO+MH group. Each rat was anesthetized using 4% chloral hydrate (0.625 mL/100g) through an intraperitoneal injection. An incision was made on the lateral side of the right tibial tubercle and a channel to the marrow cavity was built using a 1.2 mm Kirschner wire. Next, a PEEK rod (1 mm diameter, 6 mm length) coated with an established *S. aureus* biofilm (with 4.22 $\log_{10}$CFU/mm^2 of bacteria on the implant) was inserted into the cavity and the incision was closed layer by layer. One day later, 100 μL of a nanoparticle aqueous solution (2.5 mg/mL) was injected into the infection site through the existing channel, and all groups, excluding the MNP-SNO group, were treated with a 1.35 kAm^{-1}AMF for 10 min. The temperature was sustained at 50 °C. This treatment was repeated three days later.

Radiography and micro-CT scanning assessment**:** To monitor IAI progress in bone tissue, an X-ray of each rat was taken at 0, 2, and 4 weeks. Harvested tibias were also scanned using a high-resolution Micro-CT (Skyscan 1076, Skyscan, UK), and scanning results were analyzed using software provided by the manufacturer. We also acquired coronal, sagittal, and transverse sections, and corresponding 3D images, from each of the groups, and further compared the formed bone volume.

In vivo antibiofilm and anti-residual bacteria evaluation**:** After 4 weeks, all treated rats were sacrificed using an

excessive dose of injected 4% chloral hydrate, at which point the right tibia of each rat was harvested. To quantify bacterial colonies using SPM, half of these samples were soaked in liquid nitrogen and grinded into powder, which was then suspended in physiological saline and diluted ten-fold into a series of gradients. In addition, implants were retrieved from broken tibias and treated for 5 min with 40 kHz of ultrasonic concussion to detach adhered biofilms. The number of bacteria was counted using the method described above.

***Histopathological and immunohistochemical analysis*:** Tibias obtained from the four groups were decalcified in room temperature EDTA for 2 weeks, prior to being dehydrated with gradient ethanol, and embedded in paraffin. To observe the infiltration of inflammatory cells, stained bacteria, iNOS, and tissue sections were stained using H&E, Giemsa, and iNOS, followed by observing stained macrophages under an optical microscope. To confirm the degradation of MNP-SNO in vivo, eighteen implant-associated infection (IAI) models were established in the right tibias of SD rats, with injection of 100 μL MNP-SNO aqueous solutions in fifteen rats (IAI+MNP-SNO) and 100 μL physiological saline in three rats (IAI) as a control group. Three IAI rats and three IAI+MNP-SNO rats were sacrificed on the first day to harvest their tibias and other organs (hearts, livers, spleens, lungs and kidneys). Three rats treated with IAI+MNP-SNO were sacrificed on post-treatment of 7, 14, 21 and 28 days to harvest their tibias and organs, respectively. All of the tibias and other organs were corroded by aqua regia to measure the concentration of cobalt ion (Co^{2+}) with inductively coupled plasma (ICP), and the IAI group without any treatment was designed as negative control. In addition, to verify the biosafety of MNP-SNOs, organs, including hearts, livers, spleens, lungs and kidneys, were harvested from rats in each of the four groups. These organs were fixed and processed using H&E staining, after which, organ sections were observed and photographed using an optical microscope.

***Statistical analysis*:** All results were expressed as mean ± standard deviation, with differences between groups analyzed using one-way analysis of variance (ANOVA), with Tukey's multiple comparisons test. A comparison between two groups was performed by utilizing Student t-test. P values < 0.05 were considered statistically significant (*P < 0.05, **P < 0.01, ***P < 0.001, ****P < 0.0001). The software GraphPad Prism 6 (GraphPad Software, Inc., La Jolla, CA) was used for statistical analysis.

References

[1] L. R. Thurlow, M. L. Hanke, T. Fritz, A. Angle, A. Aldrich, S. H. Williams, I. L. Engebretsen, K. W. Bayles, A.

R. Horswill, T. Kielian, *J. Immunol.* **2011**, *186*, 6585.

[2] G. B. Kitchen, P. S. Cunningham, T. M. Poolman, M. Iqbal, R. Maidstone, M. Baxter, J. Bagnall, N. Begley, B. Saer, T. Hussell, L. C. Matthews, D. H. Dockrell, H. J. Durrington, J. E. Gibbs, J. F. Blaikley, A. S. Loudon, D. W. Ray, *Proc. Natl. Acad. Sci. U. S. A.* **2020**, *117*, 1543.

Supplementary Tables and Figures

Table S1 Primers used for qPCR of genes

Gene	Upper primer sequence (5' to 3')	Lower primer sequence (5' to 3')
TNF-α	TAGCCAGGAGGGAGAACAGA	CCAGTGAGTGAAAGGGACAGA
iNOS	TTGACGCTCGGAACTGTA	GTTGGTGGCATAAAGTATGTG
Arg-1	TGCTCACACTGACATCAACAC	GAGAATCCTGGTACATCTGGG
GAPDH	AAATGGTGAAGGTCGGTGTG	AGGTCAATGAAGGGGTCGTT

The primers were synthesized and provided by BioTNT.

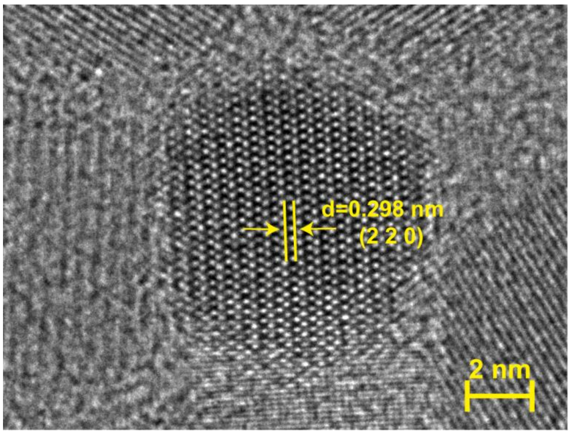

Figure S1. High-resolution TEM image of a single MNP.

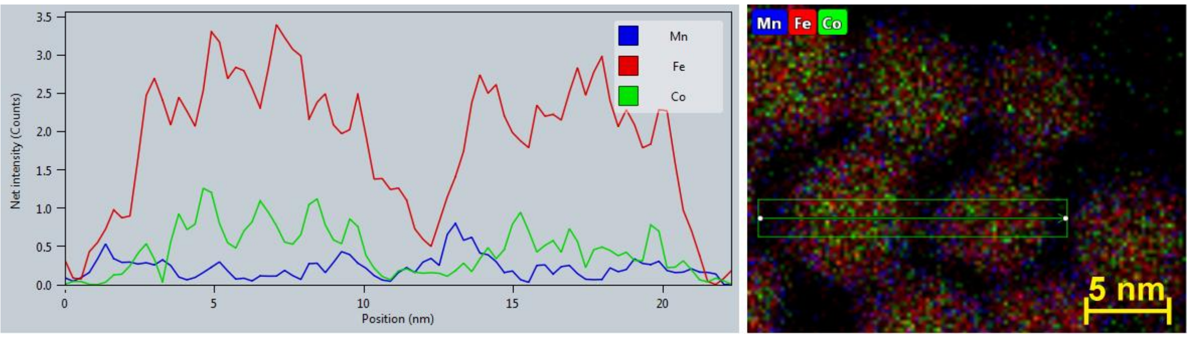

Figure S2. Co, Fe and Mn line-scanned energy-dispersive spectroscopy (EDS) profiles of MNPs.

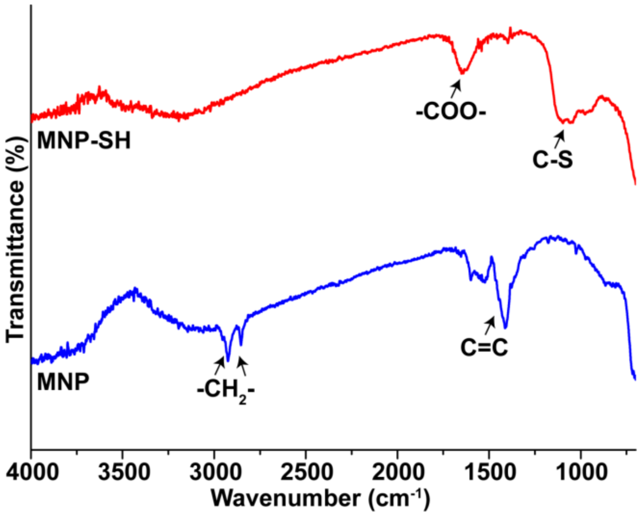

Figure S3. Infrared spectra of MNPs and MNP-SHs.

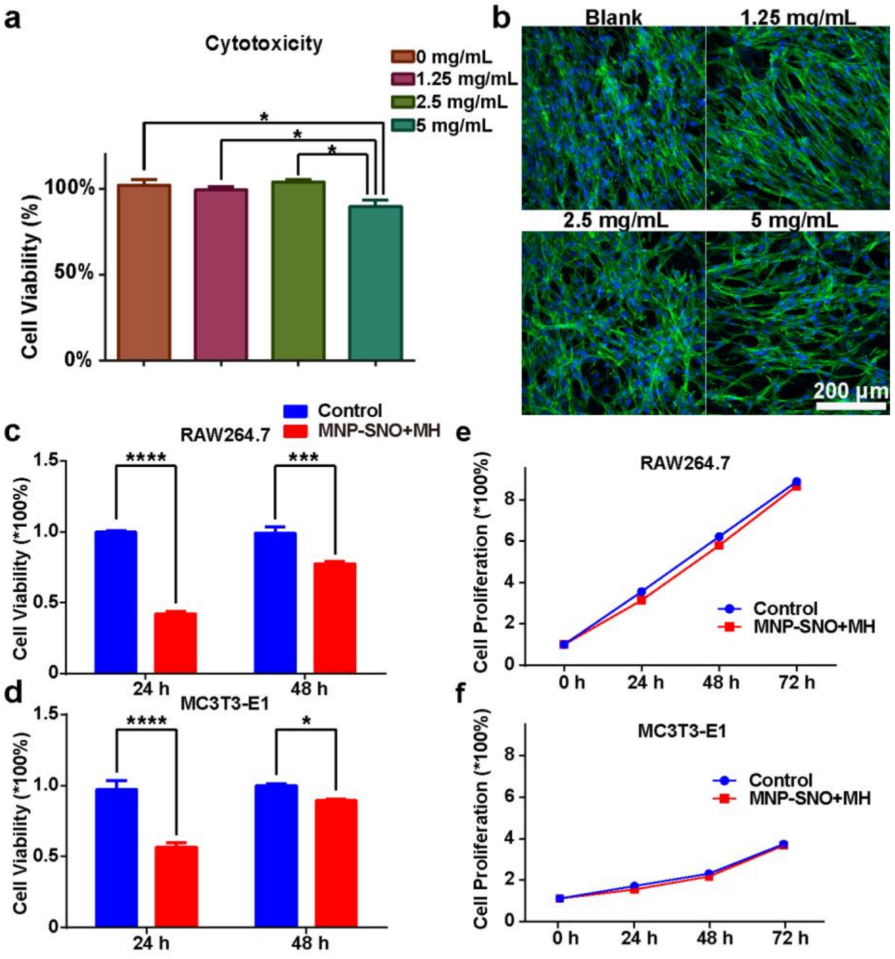

Figure S4 (a) Cell viability of HFF-1 cells cultured with MNP-SNO for 1 day (n=3, *P < 0.05) and **(b)** fluorescence images of HFF-1 cells in different concentrations of MNP-SNO for 24 h incubation. The scale bar is 200 μm. **(c)** Cell viability of RAW264.7 cells treated by MNP-SNO+magnetic hyperthermia (MH) in 24 h and 48 h (n=3, ****P < 0.0001, ***P < 0.001,); (**d**) Cell viability of MC3T3-E1 cells treated by MNP-SNO+MH in 24 h and 48 h (n=3, ****P < 0.0001, *P < 0.05 using one-way ANOVA, Tukey's multiple comparisons test); (**e**) Cell proliferation rate of survived RAW264.7 cells collected from control (treated with PBS) and MNP-SNO+MH groups at the time point of 48 h, with additional 72 h cultivation; (**f**) Cell proliferation rate of survived MC3T3-E1 cells collected from control (treated with PBS) and MNP-SNO+MH groups at the time point of 48 h, with additional 72 h cultivation.

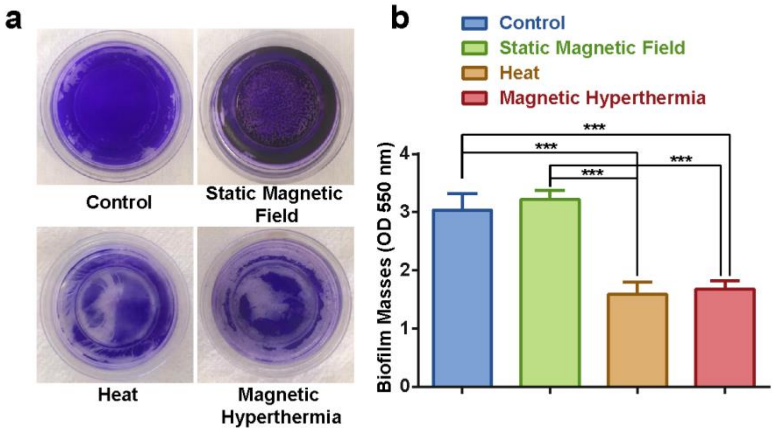

Figure S5. (a) Macroscopic biofilm images of control, static magnetic field, MNP-SH+water bathe heating and MNP-SH+MH (treated with 1.35 kAm^{-1} alternating magnetic field and NO release for 10 min) groups, stained with crystal violet. (b) Absorbance of biofilm masses at wavelength of 550 nm (n = 3, $**P < 0.01$, $***P < 0.001$ using one-way ANOVA).

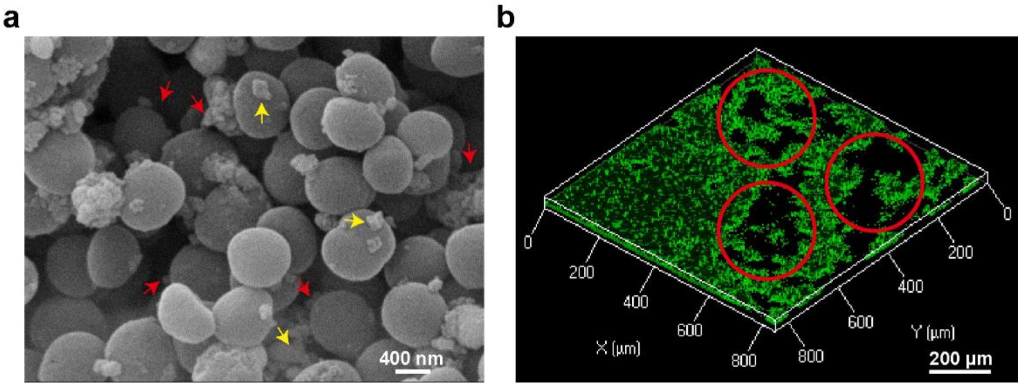

Figure S6 a) SEM image of the MNP-SNO distribution in bacterial biofilm (the yellow arrows mark the nanoparticles on the surface and the red arrows mark these nanoparticles inside the biofilms). b) The confocal microscopic image of the biofilm from MNP-SH+MH group, in which the scattered black-colored pores are clearly visible on the dissociated biofilm (the red circles indicate the pores and green area stands for all bacterial cells).

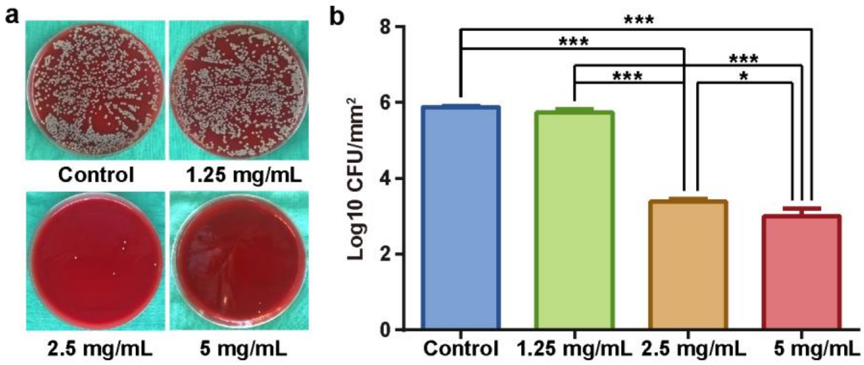

Figure S7. (a) Typical photos of *S. aureus* biofilms treated by MNP-SNO+MH with the concentration of 1.25, 2.5 and 5 mg/mL (control group: treated with saline, dilution ratio: 1:100000). (b) Counting results of *S. aureus* in groups with different concentrations by spread plate method (SPM). (n=3, $*P < 0.05$, $***P < 0.001$ using one-way ANOVA).

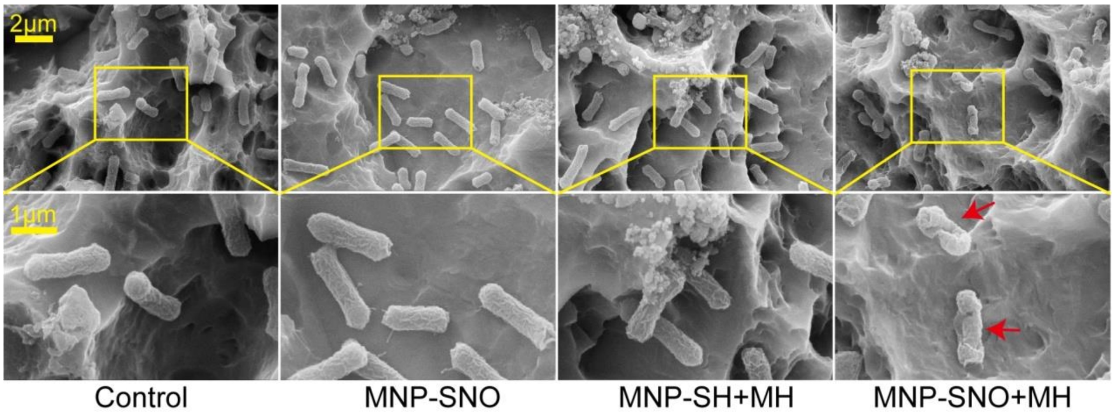

Figure S8. SEM images of *E.coli* biofilm cultured on PEEK shims and treated with saline (control group), MNP-SNO, MNP-SH+MH or MNP-SNO+MH, the red arrows point out distorted bacteria. (Scale bar: 2 μm and 1 μm)

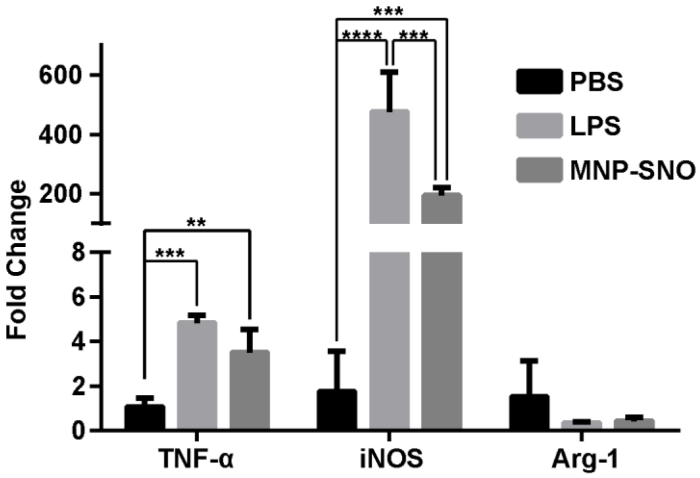

Figure S9. mRNA expression of inflammation-related genes TNF-α, iNOS and Arginase-1 (Arg-1), three groups (PBS as negative control, LPS as positive control and MNP-SNO) are included (n=3, **P < 0.01, ***P < 0.001, ****P < 0.0001 using one-way ANOVA, Tukey's multiple comparisons test).

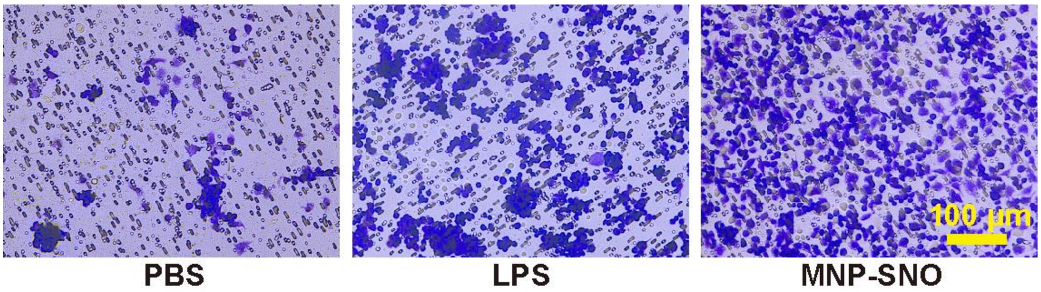

Figure S10. Images of RAW264.7 cells cultured on transwells and stimulated by PBS (negative control), LPS (positive control) or MNP-SNO, stained with crystal violet. (scale bar: 100 μm)

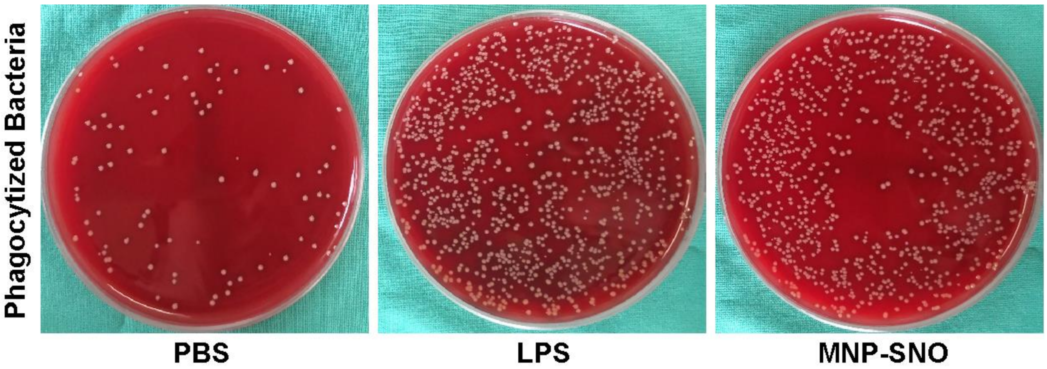

Figure S11. Representative photos of *S. aureus* phagocytized by RAW264.7 cells which were treated with PBS (negative control), LPS (positive control) or MNP-SNO (dilution ratio: 1:100).

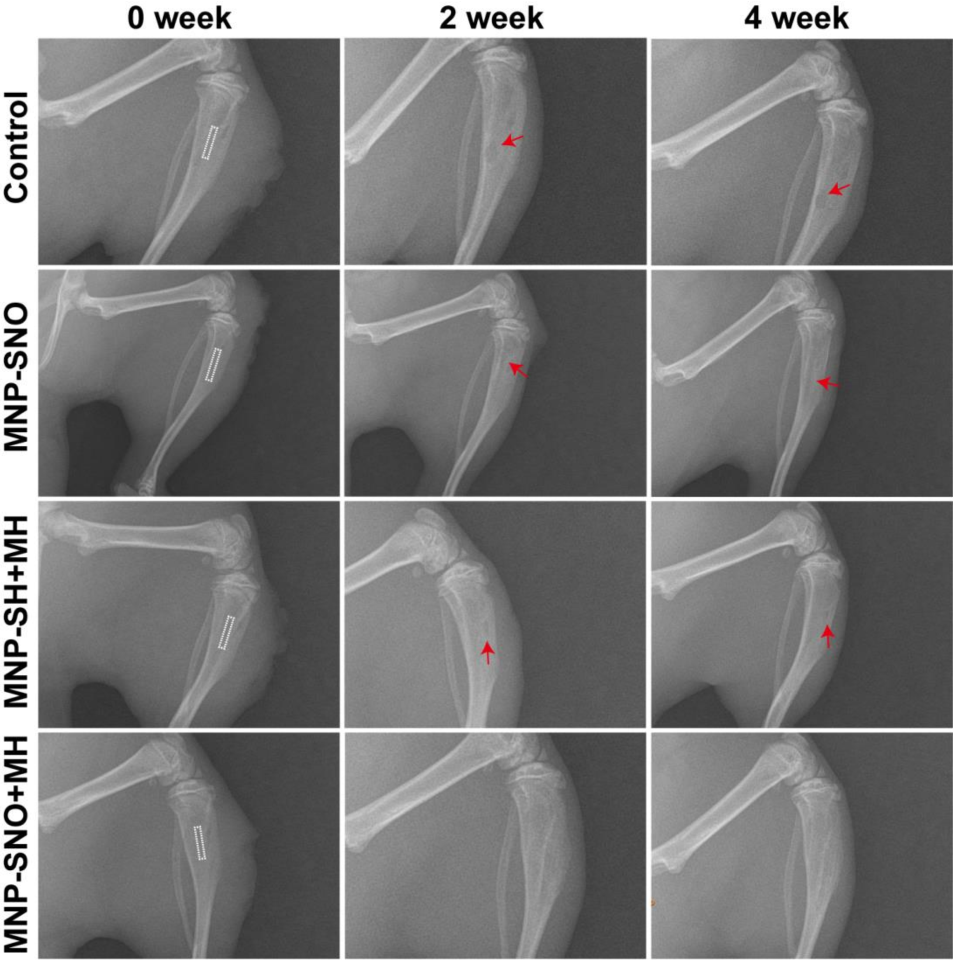

Figure S12. X-ray images of the tibias of rats at different time points (0, 2 and 4 weeks after surgery) and groups (saline as control, MNP-SNO, MNP-SH+MH and MNP-SNO+MH), the white rectangles represent inserted PEEK

rods and red arrows point out the osteolysis and infected sequestrum around the implants.

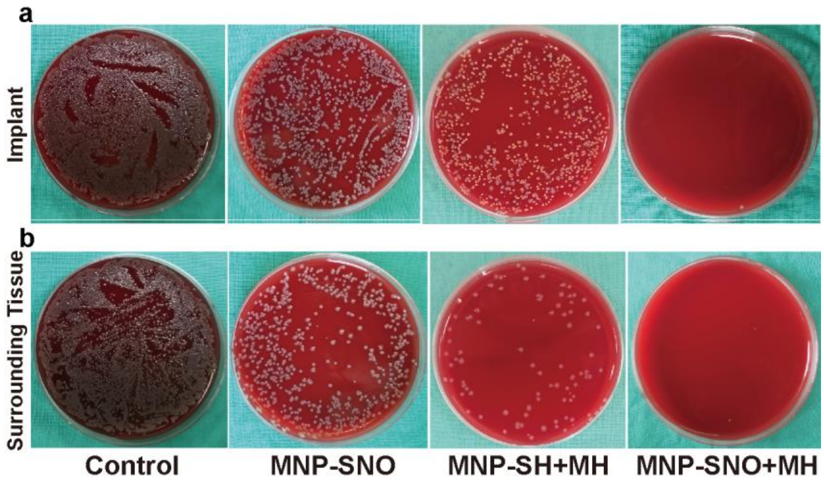

Figure S13. SPM results of biofilms detached from implants (a) and resident bacteria in surrounding tissues (b) (dilution ratio: 1:100), treatment with saline is regarded as the control group.

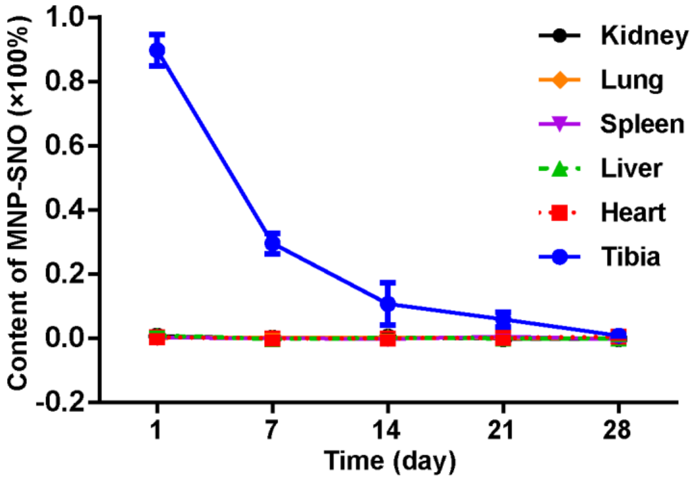

Figure S14. Degradation curve of MNP-SNO in the main organs (heart, liver, spleen, lung and kidney) and tibias at 1, 7, 14, 21 and 28 days.

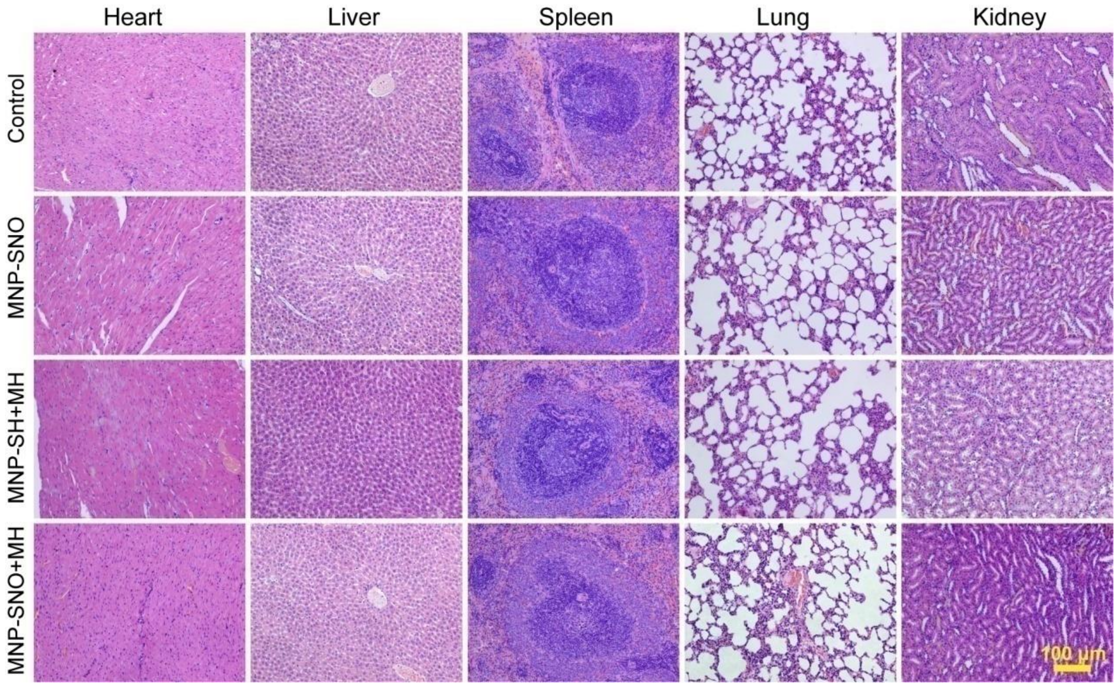

Figure S15. Histological images of H & E stained organs (hearts, livers, spleens, lungs and kidneys) harvested from different treated groups (saline as control, MNP-SNO, MNP-SH+MH and MNP-SNO+MH) (Scale bar: 100 μm).
